# Robotic-assisted TKA reduces surgery duration, length of stay and 90-day complication rate of complex TKA to the level of noncomplex TKA

**DOI:** 10.1007/s00402-022-04618-8

**Published:** 2022-10-14

**Authors:** Ricarda Stauss, Peter Savov, Lars-René Tuecking, Henning Windhagen, Max Ettinger

**Affiliations:** Department of Orthopedic Surgery, Hannover Medical School, Anna-Von-Borries-Strasse 1-7, 30625 Hanover, Germany

**Keywords:** Total knee arthroplasty, Robotic-assisted surgery, Case complexity, Readmission rate, Cost of care, Bundled payment

## Abstract

**Introduction:**

Complex primary total knee arthroplasties (TKA) are reported to be associated with excessive episode of care (EOC) costs as compared to noncomplex procedures. The impact of robotic assistance (rTKA) on economic outcome parameters in greater case complexity has not been described yet. The purpose of this study was to investigate economic outcome parameters in the 90-days postoperative EOC in robotic-assisted complex versus noncomplex procedures.

**Materials and methods:**

This study is a retrospective, single-center review of 341 primary rTKAs performed between 2017 and 2020. Patient collective was stratified into complex (*n* = 218) and noncomplex TKA (*n* = 123) based on the presence of the following criteria: Obese BMI, coronal malalignment, flexion contracture > 10°, posttraumatic status, previous correction osteotomy, presence of hardware requiring removal during surgery, severe rheumatoid arthritis. Group comparison included surgery duration, length of stay (LOS), surgical site complications, readmissions, and revision procedures in the 90-days EOC following rTKA.

**Results:**

The mean surgery duration was marginally longer in complex rTKA, but showed no significant difference (75.26 vs. 72.24 min, *p* = 0.258), neither did the mean LOS, which was 8 days in both groups (*p* = 0.605). No differences between complex and noncomplex procedures were observed regarding 90-days complication rates (7.34 vs. 4.07%, *p* = 0.227), readmission rates (3.67 vs. 3.25%, *p* = 0.841), and revision rates (2.29 vs. 0.81%, *p* = 0.318).

**Conclusions:**

Robotic-assisted primary TKA reduces the surgical time, inpatient length of stay as well as 90-days complication and readmission rates of complex TKA to the level of noncomplex TKA. Greater case complexity does not seem to have a negative impact on economic outcome parameters when surgery is performed with robotic assistance.

## Introduction

Primary total knee arthroplasty (TKA) is one of the most frequently performed elective surgical procedures and a further increase in the annual TKA-volume is predicted for the next decade [1]. Therefore, it is a major contributor to rapidly growing health care expenditures [1, 2]. The implementation of alternative payment models in health care was meant to reduce costs while maintaining a high quality of joint arthroplasty care [3]. In the bundled payment model, reimbursement is provided by a fixed payment for the financial coverage of the whole episode of care (EOC), which starts at admission and includes the 90-days postoperative episode [3, 4]. A major concern regarding this model is the lack of adequate financial adjustment for case complexity, which is associated with higher costs resulting in EOC net losses [5, 6]. In terms of arthroplasties, TKA is deemed complex if ideal component alignment is surgically challenging because of displaced anatomical landmarks and an altered mechanical axis, e.g., due to extra-articular deformities or severe coronal malalignment [7–9]. The investigation of economic outcome parameters in complex versus noncomplex manual TKA (mTKA) revealed significantly higher surgical and hospital costs as well as higher 90-days complication and readmission rates in the complex cohort [7]. Several studies identified these parameters as significant contributors to higher EOC costs; therefore, being crucial targets for potential cost reduction [10–12].

However, with the continuous improvement of surgical techniques and the implementation of new technologies, indications for primary TKA have expanded to include increasingly complex cases [13]. Evidence indicates that the robotic-assisted TKA (rTKA) increases the accuracy of bone cuts and precision of component positioning, as stated in a recent review by Elliott et al. [14–16]. Furthermore, the previous studies investigated rTKA in patients with severe coronal malalignment and extra-articular deformities concluding that rTKA is a feasible method to achieve correction to neutral alignment [17–22]. Robotic technology has been shown to have the potential to improve clinical outcomes [23], lower readmission rates and decrease EOC costs as compared to conventional TKA [24–26].

The impact of robotic assistance on economic outcome parameters in increasingly complex arthroplasties has not been described yet. With adverse cost-related outcomes being reported for complex conventional TKA, it is important to evaluate newer techniques that might have a beneficial impact on economic outcome parameters and may therefore have the potential to reduce EOC costs in greater case complexity.

The purpose of this study was to compare the surgery duration, length of stay (LOS) and the 90-days complication and readmission rates in the 90-days EOC between complex and noncomplex TKA using robotic assistance. We hypothesized that the implementation of rTKA would reduce the surgical time and the LOS in complex cases as well as the 90-days complication and readmission rates to the level of noncomplex TKA.

## Materials and methods

**Fig. 1:**
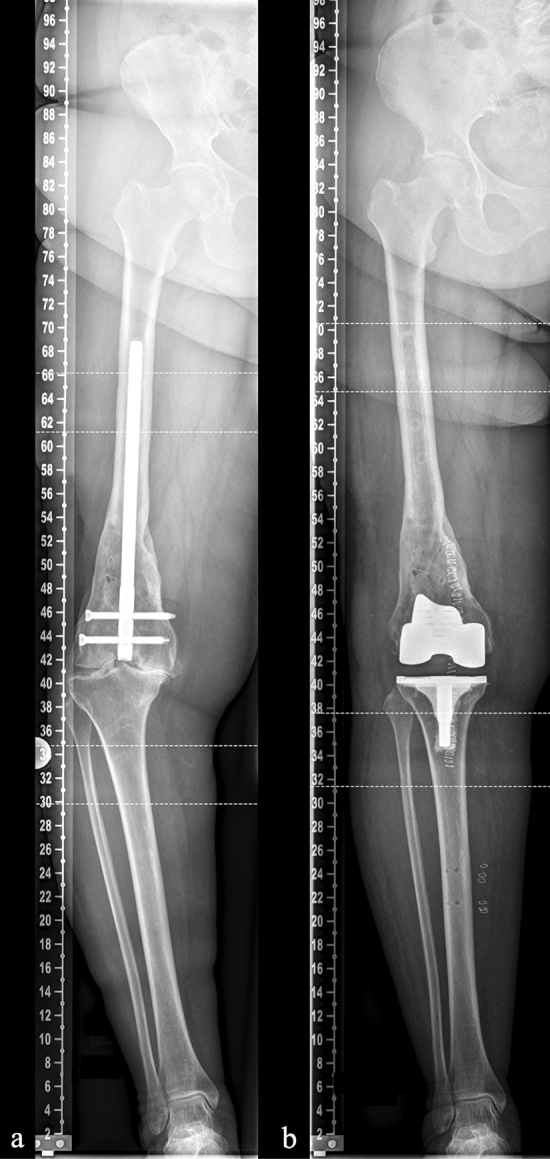
Complex rTKA in a case of post-traumatic osteoarthritis with retained hardware requiring removal during the TKA procedure. **a** Preoperative anterior − posterior weight bearing long leg view in a case of femoral fracture treated with retrograde intramedullary nailing. **b** Postoperative radiographs after rTKA

In this retrospective, single-center study, a systematic review of the institutional database was conducted to identify all robotic-assisted primary TKAs between April 2017 and December 2020. A total of 341 rTKAs were performed in 331 patients using an image-based robotic system in 182 cases (MAKO^®^, Stryker Corporation, Kalamazoo, MI, USA) and an imageless robotic system in 159 cases (NAVIO^®^, Smith and Nephew, Memphis, TN, USA). All surgeries were performed under the care of two senior surgeons. The patient collective was stratified into two groups: complex TKA (*n* = 218) and noncomplex TKA (*n* = 123). Definition of complex procedures was based on the presence of the following criteria, modified from Ryan et al. [7]: Obese BMI (> 35.68), coronal malalignment (mPTA < 83° or > 91°, lDFA < 84° or > 90°), flexion contracture > 10°, posttraumatic status, previous ipsilateral correction osteotomy, presence of hardware requiring removal during surgery, severe rheumatoid arthritis (Table 1, Fig. 1).

**Table 1:** Definition criteria for complex cases in the complex rTKA cohort

	Complex rTKA (*n* = 218)	Mean (range)
BMI > 35.68 kg/m^2^	54 (24.77)	39.52 (35.69 – 47.46)
mPTA deviation (°)		5.42 (0.6 – 16.20)
lDFA deviation (°)		4.55 (0.86 – 12.57)
Flexion contracture > 10° (°)	31 (14.22)	18.39 (15.00 – 40.00)
Posttraumatic status	17 (7.80)	
Previous correction osteotomy	27 (12.39)	
Hardware requiring removal during surgery	3 (1.37)	
Severe rheumatoid arthritis	6 (2.75)	

mPTA and lDFA deviation are defined as deviation from normal set at 87° [50]

Means and ranges are given for cases classified as complex based on this specific parameter

*BMI* body mass index, *mPTA* medial proximal tibial angle, *lDFA* lateral distal femoral angle

Demographic and clinical data were retrieved from the digital medical records including sex, age (at the time of the index procedure), body mass index (BMI), American Society of Anesthesiologists (ASA) score, comorbidities, previous surgeries, and preoperative range of motion. Intraoperative data included the robotic system, implant utilization, and surgery duration (skin incision to wound closure). Postoperative length of stay (LOS), discharge disposition, and direct postoperative complications were compared between the groups. The 90-days postoperative episode was examined regarding surgical site complications, readmission rates, and revision procedures. Arthroplasty-related postoperative complications were defined according to Iorio et al. [27] and classified as direct postoperative complications during acute inpatient stay and 90-days postoperative complications which occurred after discharge.

Pre- and postoperative radiographs included an anterior–posterior weight-bearing long leg view and a lateral view. Radiological measurements of the medial proximal tibial angle (mPTA), lateral distal femoral angle (lDFA) and overall limb alignment (OLA) were performed using Carestream PACS software (Carestream Health Deutschland GmbH, Stuttgart, Germany).

Normal distribution was tested using the Kolmogorov − Smirnov test. Group differences were calculated using Student’s *t* test for normally distributed data and Mann − Whitney *U* test for nonparametric data. Chi-square test was used to compare categorical data. A *p* value < 0.05 was considered statistically significant. Statistical analysis was performed using IBM SPSS Statistics 27 (SPSS Inc. Chicago, IL, USA).

The study was approved by the local ethics committee (#8808_BO_K_2019).

## Results

Of the 341 patients being included in this study, 192 were female (56.30%). The mean age at the time of the index procedure was 66 years, and the mean ASA score was 2. Except from a higher BMI in the complex cohort (mean 30.77 vs. 28.77, *p* < 0.001), baseline characteristics revealed no significant group differences (Table 2).

**Table 2:** Baseline characteristics for patients undergoing primary rTKA

	Complex rTKA (*n* = 218)	Noncomplex rTKA (*n* = 123)	*p* value
Age (y), mean (SD)	66.14 (SD, 9.97)	66.86 (SD, 10.32)	0.521
Sex			0.775
Male (%)	94 (43.1)	55 (44.7)	
Female (%)	124 (56.9)	68 (55.3)	
BMI (kg/m^2^), mean (SD)	30.77 (SD, 6.32)	28.77 (SD, 3.54)	< 0.001*
ASA > 2 (%)	32 (14.7)	10 (8.1)	0.077

*ASA* American Society of Anesthesiologists Score, *BMI* body mass index, *SD* standard deviation, *rTKA* robotic-assisted total knee arthroplasty

^*^Indicates statistical significance

### Surgery duration

The mean surgery duration was marginally longer in the complex rTKA cohort (75.26 vs. 72.24 min, *p* = 0.258), but did not reach statistical significance.

### Postoperative length of stay

The mean postoperative LOS was 8 days in both groups (7.76 vs. 7.60 days, *p* = 0.605) and showed no significant group difference in complex versus noncomplex rTKA patients. Patients were discharged to a type of rehabilitation facility in > 90% of all cases.

### Complication and readmission rates in complex and noncomplex rTKA

There were no significant group differences regarding the complication and readmission rates. The comparison of direct postoperative complications during acute inpatient stay revealed no significant group differences. 15 direct postoperative complications were observed in the complex and 5 in the noncomplex cohort (6.88 vs. 4.07%, *p* = 0.288), requiring one revision surgery in the complex and two in the noncomplex group (*p* = 0.268).

The 90-days complication rate was 7.34% for complex rTKA and 4.07% for standard procedures (*p* = 0.227). There were 12 readmissions in the 90-days EOC, of which 8 occurred in the complex group. Neither 90-day readmission rates (3.67 vs. 3.25%, *p* = 0.841), nor 90-day revision rates (2.29 vs. 0.81%, *p* = 0.318) differed significantly between complex and noncomplex rTKA (Table 3). Detailed information on postoperative complications is provided in Table 4.

**Table 3:** Economic outcome parameters in complex versus noncomplex rTKA

	Complex rTKA (*n* = 218)	Noncomplex rTKA (*n* = 123)	*p* value
Surgery duration (min), mean (SD)	75.26 (SD, 23.62)	72.24 (SD, 23.58)	0.258
Postoperative LOS (d), mean (SD)	7.76 (SD, 2.65)	7.60 (SD, 2.67)	0.605
Discharge disposition			0.678
Rehabilitation facility (%)	204 (93.58)	112 (91.06)	
Inpatient rehabilitation (%)	175 (80.28)	97 (78.86)	
Outpatient rehabilitation (%)	29 (13.30)	15 (12.20)	
Home (%)	14 (6.42)	11 (8.94)	
Direct postoperative complications (%)	15 (6.88)	5 (4.07)	0.288
Inpatient revision surgery (%)	1 (0.46)	2 (1.63)	0.268
90-day complications (%)	16 (7.34)	5 (4.07)	0.227
90-day readmissions (%)	8 (3.67)	4 (3.25)	0.841
90-day revision surgery (%)	5 (2.29)	1 (0.81)	0.318

*LOS* length of stay, *SD* standard deviation, *rTKA* robotic-assisted total knee arthroplasty

**Table 4:** Postoperative complications in the 90-day postoperative episode

	Complex rTKA (*n* = 218)	Noncomplex rTKA (*n* = 123)	*p* value
Wound complications (%)	6 (2.75)	0 (0.00)	0.063
Outpatient wound revision (%)	2 (0.92)	0 (0.00)	0.287
Inpatient wound revision (%)	4 (1.83)	0 (0.00)	0.131
Acute swelling, clinical signs of infection (%)	3 (1.38)	1 (0.81)	0.643
Intra-articular hematoma (%)	1 (0.46)	0 (0.00)	0.452
Joint aspiration (%)	4 (1.83)	1 (0.81)	0.451
PJI (%)	0 (0.00)	0 (0.00)	
Deep vein thrombosis	1 (1.83)	0 (0.00)	0.452
Persistent stiffness (%)	2 (0.92)	2 (1.63)	0.559
Complex conservative pain management (%)	1 (0.46)	2 (1.63)	0.268
Femoral nerve block (%)	1 (0.46)	2 (1.63)	0.268
Open arthrolysis (%)	1 (0.46)	0 (0.00)	0.452
Mechanical complications (%)	1 (0.46)	1 (0.81)	0.681
Luxation of the knee (%)	0 (0.00)	1 (0.81)	0.182
Patellar fracture (%)	0 (0.00)	1 (0.81)	0.182
Patellar luxation (%)	1 (0.46)	0 (0.00)	0.452

*PJI* Periprosthetic joint infection

## Discussion

The most important finding of the present study is that robotic-assisted TKA reduces the surgical time, postoperative length of stay as well as 90-days complication and readmission rates of complex procedures to the level of noncomplex procedures. These parameters were identified as major contributors to excessive EOC costs in previous studies and were found to be associated with greater case complexity [7, 11, 12]. As continuously rising health care costs constitute a significant economic burden, these economic outcome parameters depict crucial targets for potential cost reductions.

### Surgery duration

In contrast to Ryan et al., who found significantly prolonged surgical times for complex versus noncomplex mTKA, our investigation of robotic-assisted TKA did not prove an association between case complexity and prolonged surgery duration [7]. Cost efficiency is a major concern of rTKA and surgery duration substantially determines intraoperative costs. A prior study by Savov et al. carried out at our institution demonstrated a learning curve of 11 cases for surgical time in rTKA [28]. After completing the learning curve, there was no significant difference for surgery duration between rTKA and conventional manual TKA, which is in line with evidence from other studies [29, 30]. Registration of anatomical landmarks and the reconstruction of the patient’s individual anatomy by implant positioning and joint balancing according to soft tissue tension were identified as critical, time-consuming steps [28, 30]. With regard to greater case complexity, these are crucial for the correction of complex deformities. After completing the learning curve, most time savings were achieved in these steps. Interestingly, in the present study, mean surgery duration for complex cases was remarkably shorter than surgical time published for complex mTKA. This might indicate that in greater case complexity rTKA is capable of even providing time savings and consecutive cost savings as OR minutes depict a substantial intraoperative cost factor [31, 32]. However, this remains hypothetical and requires further studies comparing rTKA and mTKA with regard to case complexity.

### Postoperative length of stay

In this present study, mean postoperative LOS was equal for both complex and noncomplex procedures. Of note, mean LOS in this patient collective was longer than in the previous studies [2, 33], which is mainly attributable to the fact that in the German health care system patients undergoing total joint arthroplasty are regularly discharged to a rehabilitation facility. This is displayed by a discharge disposition to some type of rehabilitation facility in > 90% in this study collective. Thus, the time of discharge depends on the capacities of rehabilitation facilities and prolonged LOS is the consequence.

Our findings are in contrast to evidence from the previous studies reporting an association between greater case complexity and prolonged LOS [13, 34]. In this context, it is important to distinguish medical case complexity defined by comorbidity profiles from actual surgical case complexity. For medical case complexity, greater LOS and discharge to other destinations than home are consistently reported to be risk factors for all-cause readmissions following TKA [13, 35, 36]. This is attributable to the fact that these patients have worse overall health conditions, and are more likely to have complications or readmissions for any reasons [36]. With regard to surgical case complexity, discharge to rehabilitation facility might be even beneficial in terms of postoperative outcomes and complications, as d’Apuzzo et al. found a discharge to rehabilitation facilities to be protective against TKA-related readmissions [36].

### 90-day complication and readmission rates

In general, episode of care costs are mainly driven by higher complication and readmission rates in the 90-days postoperative episode, which often lead to costs exceeding the bundled payment [37]. Remarkably, in this present study greater case complexity was not associated with increased complication rates, readmission rates or subsequent revision procedures. The observed 90-days complication rate of 7.34% and 90-days readmission rate of 3.67% are lower than those reported by Ryan et al. for complex mTKA (15.6% and 7.8%, respectively) and in contrast to their data, this present study did not reveal significant differences as compared to noncomplex procedures [7]. Furthermore, the complication rate in this present study is consistent with evidence on 90-days surgery-related ED visits derived from register-based studies [38]. Our findings of comparable 90-days complication and readmission rates in complex and noncomplex procedures suggest that the implementation of robotic assistance in the context of increasing case complexity is capable of reducing complication rates and subsequent utilization of the healthcare resources to the level of standard procedures.

### Robotic assistance in surgical case complexity

In the present study, TKA surgery was performed using two different robotic systems. With the imageless system, the virtual model of the knee is created by intraoperative mapping of the patient’s osseous structures [39]. Component size and implant positioning are determined intraoperatively and bone resection is performed using a handheld robotic burr [39]. With the image-based system, the surgical procedure is preplanned based on the preoperative imaging and bone resection is performed with a robotic-arm saw system [40, 41]. These differences indicate that there are slightly different intraoperative workflows. On the other hand, both systems are semi-active robotic systems and provide a broad range of common features. Both systems calculate a three-dimensional virtual model of the patient’s unique anatomy which enables the surgeon to achieve accurate bone resection limited within the confines of the surgical plan and optimal implant positioning with regard to the patient’s individual features [16, 40]. Both robotic systems provide real-time intraoperative data and thereby help the surgeon to achieve optimal results as component positioning, alignment, balanced flexion − extension gaps, proper soft tissue tension, and range of motion can be validated intraoperatively [16, 40, 41].

It is known that rTKA in general is capable of achieving higher precision of component positioning by intraoperative three-dimensional implant planning, which takes the patient’s individual anatomy into account [15]. This is also advantageous for the correction of complex deformities as the intraoperative plan provides an appropriate assessment of the deformity and allows for the best possible correction to neutral with exact bone cuts and minimal soft tissue releases [22]. Prior studies investigated rTKA in severe coronal deformities (post-traumatic) extra-articular deformities, flexion contracture and retained hardware [17, 18, 21, 22, 42]. The results are promising in terms of component positioning, gap balancing, restoration of the mechanical axis and improved function [9]. These factors are crucial for the long-term survivorship of TKA since postoperative malalignment can lead to early failure of TKA [9, 43].

Several studies focused on medical comorbidity profiles and patient characteristics associated with higher EOC costs [6, 13, 44–47], but there is a lack of data on actual surgical case complexity and subsequent arthroplasty-related complications. This is surprising, as the implementation of new technologies has the potential to optimize TKA surgery even in complex cases. The findings of this present study add on to the benefits of robotic assistance in complex cases, as not only surgical time and postoperative length of stay, but also the 90-day complication, readmission and revision rates for complex procedures were reduced to the same level as noncomplex rTKA procedures.

Concerns remain regarding the cost-effectiveness of rTKA, as increased costs are linked to the initial purchase and maintenance of the system [9, 48]. On the other hand, there is a potential for cost savings due to decreased downstream costs, e.g. due to reduced utilization of healthcare resources in the 90-day EOC. Several studies suggest that increased expenditures may be offset by improved postoperative outcomes, implant − survival rates and decreased revision rates [26, 31, 48, 49], but long-term outcomes remain to be demonstrated.

## Limitations

This study has some limitations that are worth noting. First, the study population is limited since this is a single-center study based on our institutional database only. Owing to the retrospective design, no clinical data is presented. However, this is offset by the fact, that patients are encouraged to return to the outpatient clinic for a standardized 3-months follow-up visit, so information on the 90-days postoperative EOC is covered. Furthermore, in most cases TKA-related complications are readmitted to the primary institution.

In contrast to prior studies, we did not focus on the aspect of medical case complexity. However, we assessed the preoperative ASA score as a parameter for medically complex cases, which revealed no significant group difference.

Inpatient length of stay is not a standardized parameter as there are substantial differences in international health care systems due to different discharge protocols, which makes an absolute count of days difficult to compare to evidence from other countries. Owing to the retrospective study design, the exact time when a patient met discharge criteria could not be determined reliably, thus this limitation related to the German system could not be ruled out.

This study does not include explicit data on EOC costs. The aim of the present study was to give a first overview of the impact of robot-assisted technologies on economic outcome parameters in greater case complexity and provide general findings that are applicable to other institutions and countries. Because no significant group differences were observed for any of the outcome parameters, there is little reason to believe that the incorporation of cost data would alter these general findings. Nevertheless, further studies are necessary to corroborate the general findings of this study with cost data.

## Conclusion

Despite these limitations, this study is to our best knowledge the first to investigate the impact of rTKA on economic outcome parameters in surgical case complexity. In conclusion, robotic-assisted TKA shows promising results for complex primary TKA in terms of surgery duration, postoperative length of stay, complication rates and subsequent utilization of healthcare resources in the 90-days EOC which are reduced to the level of noncomplex procedures. Therefore, the implementation of robotic assistance may be a promising approach to address these economic outcome parameters as crucial targets for potential cost savings in the 90-days EOC.

## References

[1] Kurtz S, Ong K, Lau E, Mowat F, Halpern M (2007). Projections of primary and revision hip and knee arthroplasty in the United States from 2005 to 2030. J Bone Joint Surg Am.

[2] Cram P, Lu X, Kates SL, Singh JA, Li Y, Wolf BR (2012). Total knee arthroplasty volume, utilization, and outcomes among Medicare beneficiaries, 1991–2010. JAMA.

[3] Froimson MI, Rana A, White RE, Marshall A, Schutzer SF, Healy WL (2013). Bundled payments for care improvement initiative: the next evolution of payment formulations: AAHKS Bundled Payment Task Force. J Arthroplasty.

[4] No authors listed. Bundled Payments for Care Improvement (BPCI) Initiative: General Information. https://innovation.cms.gov/innovation-models/bundled-payments. Accessed 01 Mai 2021

[5] Kamath AF, Courtney PM, Bozic KJ, Mehta S, Parsley BS, Froimson MI (2015). Bundled payment in total joint care: survey of AAHKS membership attitudes and experience with alternative payment models. J Arthroplasty.

[6] Anis HK, Sodhi N, Vakharia RM, Scuderi GR, Malkani AL, Roche MW (2021). Cost analysis of medicare patients with varying complexities who underwent total knee arthroplasty. J Knee Surg.

[7] Ryan SP, Wu CJ, Plate JF, Bolognesi MP, Jiranek WA, Seyler TM (2021). A case complexity modifier is warranted for primary total knee arthroplasty. J Arthroplasty.

[8] Baldini A, Castellani L, Traverso F, Balatri A, Balato G, Franceschini V (2015). The difficult primary total knee arthroplasty: a review. Bone Joint J.

[9] Ross KA, Wiznia DH, Long WJ, Schwarzkopf R (2021). The use of computer navigation and robotic technology in complex total knee arthroplasty. JBJS Rev.

[10] Lygrisse KA, Zak S, Singh V, Hutzler LH, Schwarzkopf R, Rozell JC (2021). Emergency department observation versus readmission following total joint arthroplasty: Can we avoid the bundle buster?. J Arthroplasty.

[11] Clair AJ, Evangelista PJ, Lajam CM, Slover JD, Bosco JA, Iorio R (2016). Cost analysis of total joint arthroplasty readmissions in a bundled payment care improvement initiative. J Arthroplasty.

[12] Phillips JLH, Rondon AJ, Vannello C, Fillingham YA, Austin MS, Courtney PM (2019). How much does a readmission cost the bundle following primary hip and knee arthroplasty?. J Arthroplasty.

[13] Anis HK, Sodhi N, Acuna AJ, Roth A, Vakharia R, Newman JM (2020). Does increasing patient complexity have an effect on medical outcomes and lengths-of-stay after total knee arthroplasty?. J Knee Surg.

[14] Hampp EL, Chughtai M, Scholl LY, Sodhi N, Bhowmik-Stoker M, Jacofsky DJ (2019). Robotic-arm assisted total knee arthroplasty demonstrated greater accuracy and precision to plan compared with manual techniques. J Knee Surg.

[15] Khlopas A, Sodhi N, Sultan AA, Chughtai M, Molloy RM, Mont MA (2018). Robotic arm-assisted total knee arthroplasty. J Arthroplasty.

[16] Elliott J, Shatrov J, Fritsch B, Parker D (2021). Robotic-assisted knee arthroplasty: an evolution in progress. A concise review of the available systems and the data supporting them. Arch Orthop Trauma Surg.

[17] Marchand RC, Sodhi N, Khlopas A, Sultan AA, Higuera CA, Stearns KL (2018). Coronal correction for severe deformity using robotic-assisted total knee arthroplasty. J Knee Surg.

[18] Marchand RC, Khlopas A, Sodhi N, Condrey C, Piuzzi NS, Patel R (2018). Difficult cases in robotic arm-assisted total knee arthroplasty: a case series. J Knee Surg.

[19] Bottros J, Klika AK, Lee HH, Polousky J, Barsoum WK (2008). The use of navigation in total knee arthroplasty for patients with extra-articular deformity. J Arthroplasty.

[20] Chou WY, Ko JY, Wang CJ, Wang FS, Wu RW, Wong T (2008). Navigation-assisted total knee arthroplasty for a knee with malunion of the distal femur. J Arthroplasty.

[21] Catani F, Digennaro V, Ensini A, Leardini A, Giannini S (2012). Navigation-assisted total knee arthroplasty in knees with osteoarthritis due to extra-articular deformity. Knee Surg Sports Traumatol Arthrosc.

[22] Klein GR, Austin MS, Smith EB, Hozack WJ (2006). Total knee arthroplasty using computer-assisted navigation in patients with deformities of the femur and tibia. J Arthroplasty.

[23] Shearman AD, Sephton BM, Wilson J, Nathwani DK (2021). Robotic-assisted unicompartmental knee arthroplasty is associated with earlier discharge from physiotherapy and reduced length-of-stay compared to conventional navigated techniques. Arch Orthop Trauma Surg.

[24] Kayani B, Konan S, Tahmassebi J, Pietrzak JRT, Haddad FS (2018). Robotic-arm assisted total knee arthroplasty is associated with improved early functional recovery and reduced time to hospital discharge compared with conventional jig-based total knee arthroplasty: a prospective cohort study. Bone Joint J.

[25] Grosso MJ, Li WT, Hozack WJ, Sherman M, Parvizi J, Courtney PM (2020). Short-term outcomes are comparable between robotic-arm assisted and traditional total knee arthroplasty. J Knee Surg.

[26] Mont MA, Cool C, Gregory D, Coppolecchia A, Sodhi N, Jacofsky DJ (2021). Health care utilization and payer cost analysis of robotic arm assisted total knee arthroplasty at 30, 60, and 90 days. J Knee Surg.

[27] Iorio R, Della Valle CJ, Healy WL, Berend KR, Cushner FD, Dalury DF (2014). Stratification of standardized TKA complications and adverse events: a brief communication. Clin Orthop Relat Res.

[28] Savov P, Tuecking LR, Windhagen H, Ehmig J, Ettinger M (2021). Imageless robotic handpiece-assisted total knee arthroplasty: a learning curve analysis of surgical time and alignment accuracy. Arch Orthop Trauma Surg.

[29] Sodhi N, Khlopas A, Piuzzi NS, Sultan AA, Marchand RC, Malkani AL (2018). The learning curve associated with robotic total knee arthroplasty. J Knee Surg.

[30] Kayani B, Konan S, Huq SS, Tahmassebi J, Haddad FS (2019). Robotic-arm assisted total knee arthroplasty has a learning curve of seven cases for integration into the surgical workflow but no learning curve effect for accuracy of implant positioning. Knee Surg Sports Traumatol Arthrosc.

[31] Cotter EJ, Wang J, Illgen RL (2020). Comparative cost analysis of robotic-assisted and jig-based manual primary total knee arthroplasty. J Knee Surg.

[32] Cerha O, Kirschner S, Gunther KP, Lutzner J (2009). Cost analysis for navigation in knee endoprosthetics. Orthopade.

[33] Oh C, Slover JD, Bosco JA, Iorio R, Gold HT (2018). Time trends in characteristics of patients undergoing primary total hip and knee arthroplasty in California, 2007–2010. J Arthroplasty.

[34] Shah A, Memon M, Kay J, Wood TJ, Tushinski DM, Khanna V (2019). Preoperative patient factors affecting length of stay following total knee arthroplasty: a systematic review and meta-analysis. J Arthroplasty.

[35] Nichols CI, Vose JG (2016). Clinical outcomes and costs within 90 days of primary or revision total joint arthroplasty. J Arthroplasty.

[36] D'Apuzzo M, Westrich G, Hidaka C, Jung Pan T, Lyman S (2017). All-cause versus complication-specific readmission following total knee arthroplasty. J Bone Joint Surg Am.

[37] Bosco JA, Karkenny AJ, Hutzler LH, Slover JD, Iorio R (2014). Cost burden of 30-day readmissions following Medicare total hip and knee arthroplasty. J Arthroplasty.

[38] Kelly MP, Prentice HA, Wang W, Fasig BH, Sheth DS, Paxton EW (2018). Reasons for ninety-day emergency visits and readmissions after elective total joint arthroplasty: results from a US integrated healthcare system. J Arthroplasty.

[39] Bautista M, Manrique J, Hozack WJ (2019). Robotics in total knee arthroplasty. J Knee Surg.

[40] Kayani B, Haddad FS (2019). Robotic total knee arthroplasty: clinical outcomes and directions for future research. Bone Joint Res.

[41] Roche M (2021). The MAKO robotic-arm knee arthroplasty system. Arch Orthop Trauma Surg.

[42] Matassi F, Cozzi Lepri A, Innocenti M, Zanna L, Civinini R, Innocenti M (2019). Total knee arthroplasty in patients with extra-articular deformity: restoration of mechanical alignment using accelerometer-based navigation system. J Arthroplasty.

[43] Jones CW, Jerabek SA (2018). Current role of computer navigation in total knee arthroplasty. J Arthroplasty.

[44] Goltz DE, Ryan SP, Howell CB, Attarian D, Bolognesi MP, Seyler TM (2019). A weighted index of elixhauser comorbidities for predicting 90-day readmission after total joint arthroplasty. J Arthroplasty.

[45] Karas V, Kildow BJ, Baumgartner BT, Green CL, Attarian DE, Bolognesi MP (2018). Preoperative patient profile in total hip and knee arthroplasty: predictive of increased Medicare payments in a bundled payment model. J Arthroplasty.

[46] Ponnusamy KE, Marsh JD, Somerville LE, McCalden RW, Vasarhelyi EM (2018). Ninety-day costs, reoperations, and readmissions for primary total knee arthroplasty patients with varying body mass index levels. J Arthroplasty.

[47] Ryan SP, Goltz DE, Howell CB, Jiranek WA, Attarian DE, Bolognesi MP (2019). Predicting costs exceeding bundled payment targets for total joint arthroplasty. J Arthroplasty.

[48] Novak EJ, Silverstein MD, Bozic KJ (2007). The cost-effectiveness of computer-assisted navigation in total knee arthroplasty. J Bone Joint Surg Am.

[49] Gothesen O, Slover J, Havelin L, Askildsen JE, Malchau H, Furnes O (2013). An economic model to evaluate cost-effectiveness of computer assisted knee replacement surgery in Norway. BMC Musculoskelet Disord.

[50] MacDessi SJ, Griffiths-Jones W, Harris IA, Bellemans J, Chen DB (2021). Coronal Plane Alignment of the Knee (CPAK) classification. Bone Joint J.

